# Pain management beyond opioids: a β-arrestin2-biased allosteric GPCR modulator opens new avenues for drug development

**DOI:** 10.1038/s41392-025-02361-1

**Published:** 2025-08-27

**Authors:** Eline Pottie, Sophie A. M. Steinmüller, Michael Decker

**Affiliations:** 1 Laboratory of Toxicology, Department of Bioanalysis, Faculty of Pharmaceutical Sciences, Ghent University, Ghent, Belgium; 2 School of Medicine, Medical Sciences and Nutrition, Institute of Medical Sciences, University of Aberdeen Foresterhill, Aberdeen, UK; 3 Pharmazeutische und Medizinische Chemie, Institut für Pharmazie und Lebensmittelchemie, Julius-Maximilians-Universität Würzburg (JMU), Würzburg, Germany

**Keywords:** Target validation, Drug development

A remarkable study by Guo et al., published in *Cell*, suggests a compelling new direction for improving pain management: biased allosteric modulation of the neurotensin receptor 1 (NTSR1), using the drug-like molecule SBI-810, promotes β-arrestin2 (βarr2) recruitment while avoiding canonical G protein signaling – thereby providing robust analgesia across a plethora of rodent models of both acute and chronic pain without impairing motor function, cognition, or causing opioid-like dependency.^1,2^ SBI-810 is introduced as a highly promising molecule underscoring the therapeutic potential of biased and allosteric G protein-coupled receptor (GPCR) ligands to address an urgent unmet medical need.

Allosteric modulators and biased GPCR ligands are hot topics in GPCR research. While allosteric modulation has unveiled new dimensions of receptor signaling,^3^ it remains unclear to what degree biased ligands will alter pharmacological response in a physiological setting. Opioids remain the most effective and established treatment for moderate to severe pain. Yet, their clinical success is undermined by common adverse effects: tolerance, dependence, respiratory depression, constipation, withdrawal symptoms, and high abuse potential. These consequences, exacerbated by wide-spread prescription of opioids, have contributed to a global epidemic of misuse and overdose.^4^ Attempts to engineer safer opioid drugs include the design of G protein-biased µ-opioid receptor agonists for maintaining analgesia while minimizing βarr-mediated side effects.^1,4^ However, the most promising such agent with improved safety profiles, oliceridine, seems to retain some of the liabilities of its predecessors.^1,4^ Thus, the search for non-opioid analgesics offering pain relief without opioid-associated risks is urgent.

Non-opioid analgesics like gabapentin and suzetrigine (VX-548; a recently FDA-approved NaV1.8 inhibitor) avoid opioid-related side effects, but their therapeutic applicability is mainly limited to neuropathic pain and diabetic polyneuropathy. Hence, while non-GPCR targets such as voltage-gated sodium channels (e.g., NaV1.8) and pronociceptive *N*-methyl-*D*-aspartate receptors (NMDARs) have been explored, the requirement to target both the peripheral and central nervous system (PNS and CNS) is demanding more integrative approaches.^1,4^ Unlike VX-548’s peripheral mechanism, SBI-810 engages both PNS and CNS targets, suggesting broader therapeutic potential.

Here, the unique pharmacology of SBI-810 unveils a distinct antinociceptive pathway via allosterically targeting the (previously described pain target receptor) NTSR1, that not only circumvents opioid pathways but also enhances opioid-induced analgesia. Employing an extensive panel of in vitro and in vivo studies, Guo et al. thoroughly characterized the analgesic properties of SBI-810 and introduced a previously unexploited mechanism tackling both acute and chronic pain by addressing just one GPCR. Firstly, SBI-810 was able to effectively mitigate acute pain in naïve mice under basal physiological conditions, without impairing motor function, tactile sensation, or blood flow. Secondly, the application of multiple mouse models of pain revealed that SBI-810 alleviates postoperative, neuropathic, and inflammatory pain, and showed greater efficacy than gabapentin at reducing spared nerve injury-induced mechanical pain (neuropathic pain model), without adverse side effects like sedation or cognitive impairment. Moreover, in a bone fracture model, SBI-810 relieved mechanical pain more strongly than the G protein-biased opioid analgesic oliceridine. Remarkably, while SBI-810’s antinociceptive actions are not mediated by the endogenous opioid system, it enhances opioid-induced analgesia. Moreover, the compound was found to counteract chronic opioid-induced tolerance and alleviate early stages of morphine withdrawal symptoms, without being addictive on its own. Taken together, these data points to SBI-810 as being a remarkably effective pain-ameliorating molecule lacking severe side-effects typically observed with opioid use. While these findings are encouraging, comprehensive toxicological profiling and pharmacokinetic characterization of SBI-810 remain essential to fully assess its clinical translation potential and address potential developmental challenges.

From a pharmacological perspective, the authors introduce an exciting new mechanism of pain modulation. Due to central sensitization in widespread chronic pain, the key to effective treatment may involve both the PNS and CNS. It had previously been established that βarr activity is beneficial in NMDAR-mediated analgesia.^1^ However, employing a βarr2-biased allosteric modulator to selectively target the NTSR1, which thereby indirectly regulates closely colocalized peripheral sodium channels (NaV1.7) and central ionotropic receptors (NMDAR GluN2B), adds a well-thought-out layer to the approach.

**Fig. 1:**
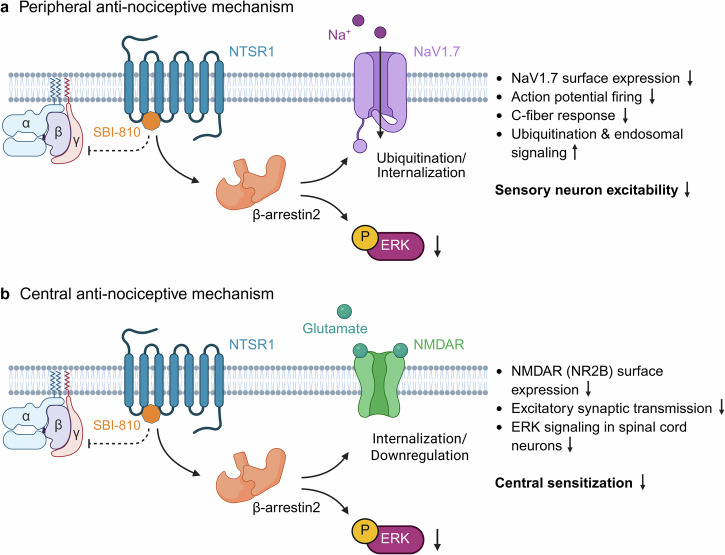
Schematic representation of the peripheral and central anti-nociceptive mechanisms mediated by SBI-810 selectively targeting NTSR1. **a** In the PNS, nociception is caused by increased sensory neuron excitability. Mechanistically, SBI-810 causes βarr2 recruitment to the NTSR1, which in turn induces internalization of NaV1.7, thereby reducing its cell surface expression in DRG neurons. Further, βarr2 may scaffold E3 ligases to promote increased ubiquitination of NaV1.7 via SBI-810. At cellular level, SBI-810 also inhibited formalin-induced ERK phosphorylation (pERK), which regulates peripheral sensitization via NaV1.7. Together, this mechanism decreased action potentials, C-fiber response and sensory neuron excitability. **b** In the CNS, pain is mediated by altered neurotransmission causing central sensitization, a mechanism involving crosstalk between βarr2, NMDARs, and ERK. SBI-810-mediated βarr2 recruitment to the NTSR1 leads to a reduction in NMDAR GluN2B surface expression, most likely through βarr2-mediated internalization. While NMDARs can drive chronic pain via pERK, SBI-810 blocked NMDA-induced pERK in spinal cord neurons. Combined, this mechanism reduced neuronal excitability and reduced excitatory postsynaptic currents. Created with BioRender.com

Specifically, the observed analgesic effect was shown to depend on NTSR1 and βarr2, but not on NTSR2 or βarr1, employing rodent models of NTSR1^−/−^ or NTSR2^−/−^ and βarr1^−/−^ or βarr2^−/−^ mice. Given that endogenous NT is essential for SBI-810-induced analgesia, as evidenced by in vivo combination studies showing that neither SBI-810 nor NT alone had an effect on paw withdrawal thresholds, SBI-810’s effects can be considered highly selective. Due to NTSR1 localization in the CNS and PNS, both peripheral and central nociceptive mechanisms are involved in the diverse analgesic actions (illustrated in Fig. 1). Interestingly, NTSR1 does not directly contribute to nociceptive modulation through its own signaling. Instead, it functions as a βarr2 recruitment platform - activated by SBI-810, and ultimately, the effects are enabled by close colocalization with sodium channel NaV1.7 in the PNS and NMDAR GluN2B in the CNS (Overview depicted in Fig. 1).

At the peripheral level, pain involves sensitization through increased sensory neuron excitability. Here, SBI-810 causes an array of antinociceptive effects: (i) βarr2-mediated internalization and ubiquitination of NaV1.7, (ii) reduced C-fiber responses (an effect lacking in NTSR1^−/−^ mice), and (iii) decreased firing rate of action potentials in dorsal root ganglia (DRG) neurons, which combinatorially induce sustained antinociception. The inhibitory effects of SBI-810 on neuronal excitability and action potential firing are retained in human DRG neurons, showing promise for the translatability of the obtained outcomes. In the CNS, the study indicates a crosstalk between NTSR1 and βarr2 for the regulation of central sensitization via NMDAR and extracellular signal-regulated kinases (ERK). SBI-810 interferes with this axis by multiple mechanisms: (i) βarr2-mediated reduction of NMDAR GluN2B surface expression, (ii) inhibition of NMDAR N2B-induced (and to a lesser extent N2A) ERK signaling in spinal cord neurons, and (iii) reduction of excitatory synaptic transmission.

Overall, SBI-810 clearly demonstrates the potential of both, pathway-specific drugs, and allosteric modulators - as well as their combined action. Historically, most allosteric modulators and biased ligands have been discovered by serendipity. Broad pharmacological characterization across multiple signaling pathways remains essential to identify desired effects and compounds. Advances like the GPCR pocketome reveal untapped allosteric sites,^5^ offering valuable starting points for novel allosteric modulators - and biased ligands. In the case of SBI-810, toxicological profiles and pharmacokinetic parameters have not been fully optimized yet. Given the steep structure-activity relationships typical of allosteric ligands, where minor chemical changes can completely alter pharmacological profiles, refinements for pharmacokinetics - necessary in drug development - must be carefully balanced. Further clinical studies will show if the substantial promise of this compound holds true. Overall, this highlights the uniqueness of SBI-810’s in vitro and in vivo actions, and its potential of opening a new avenue in drug development.
